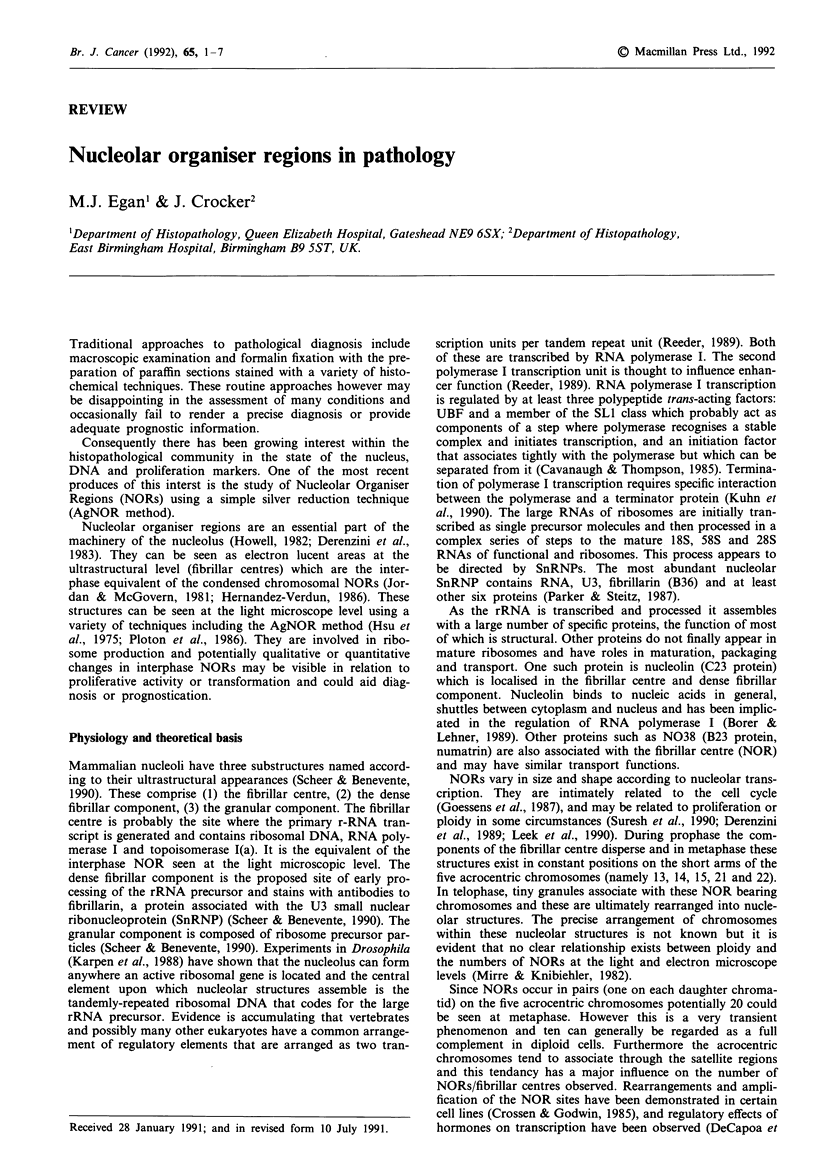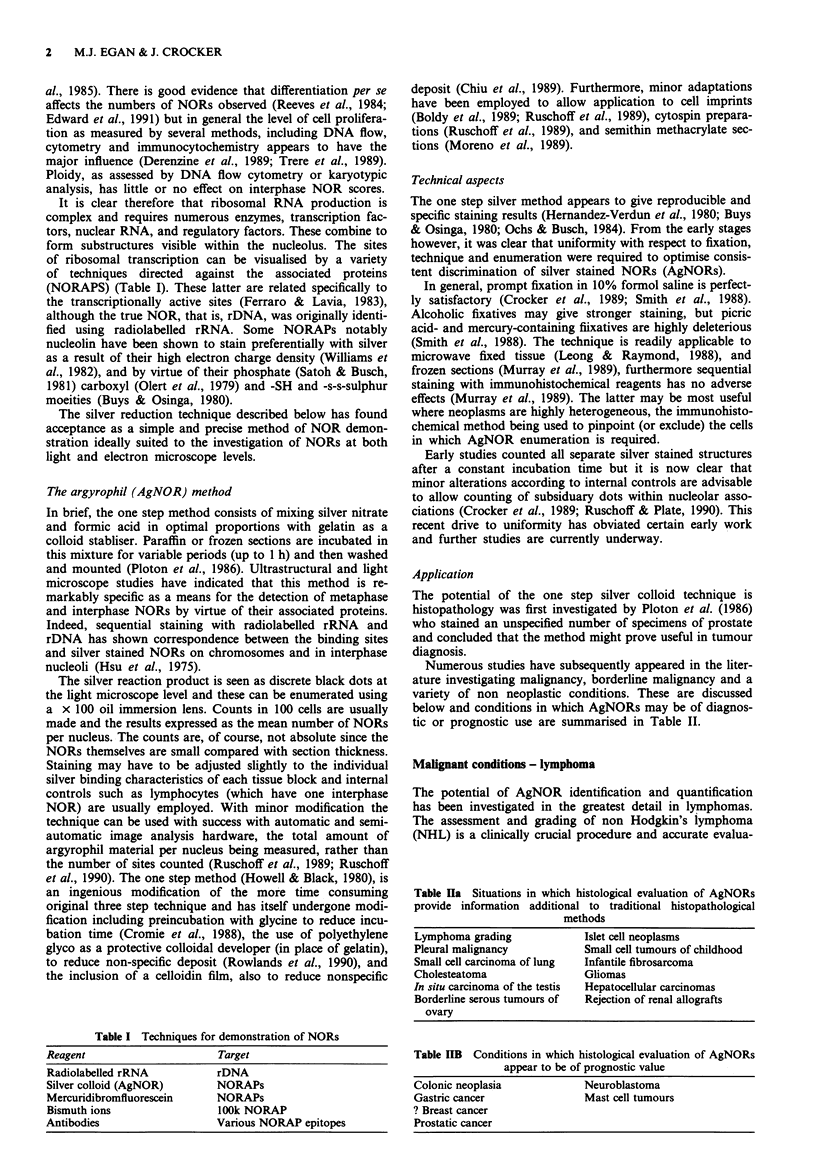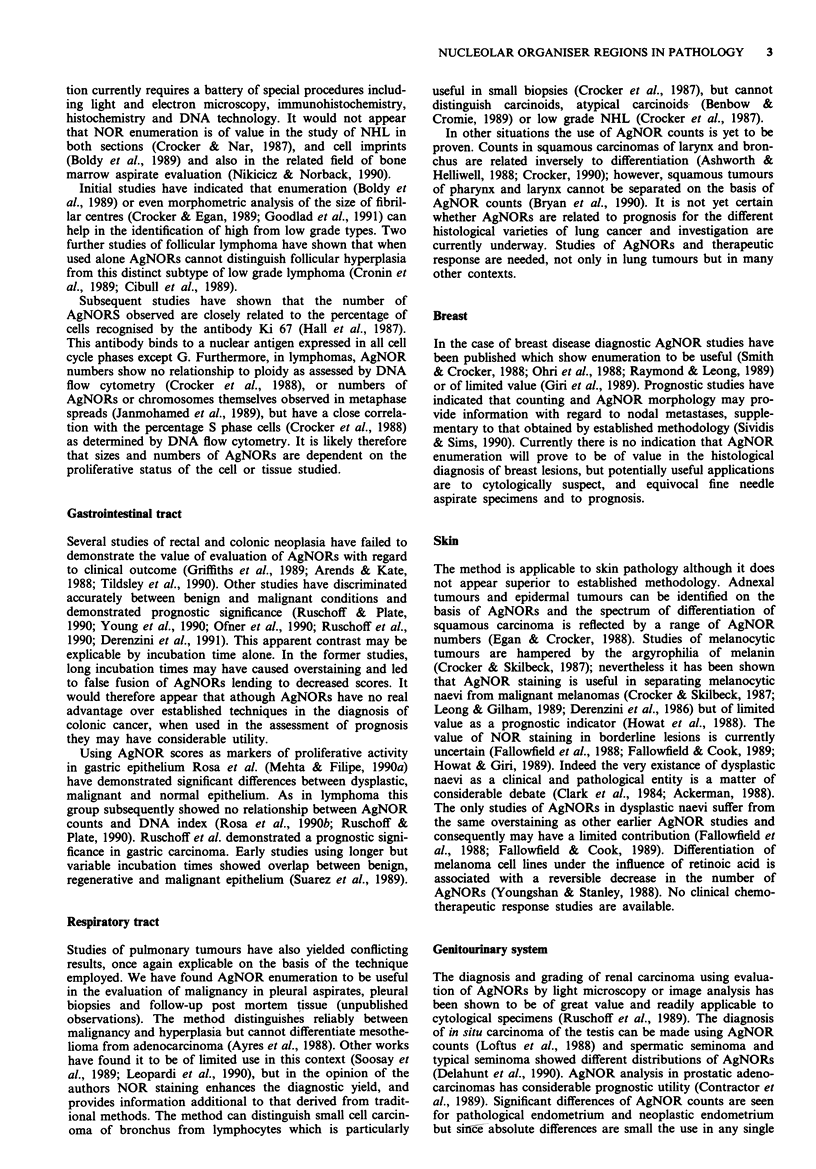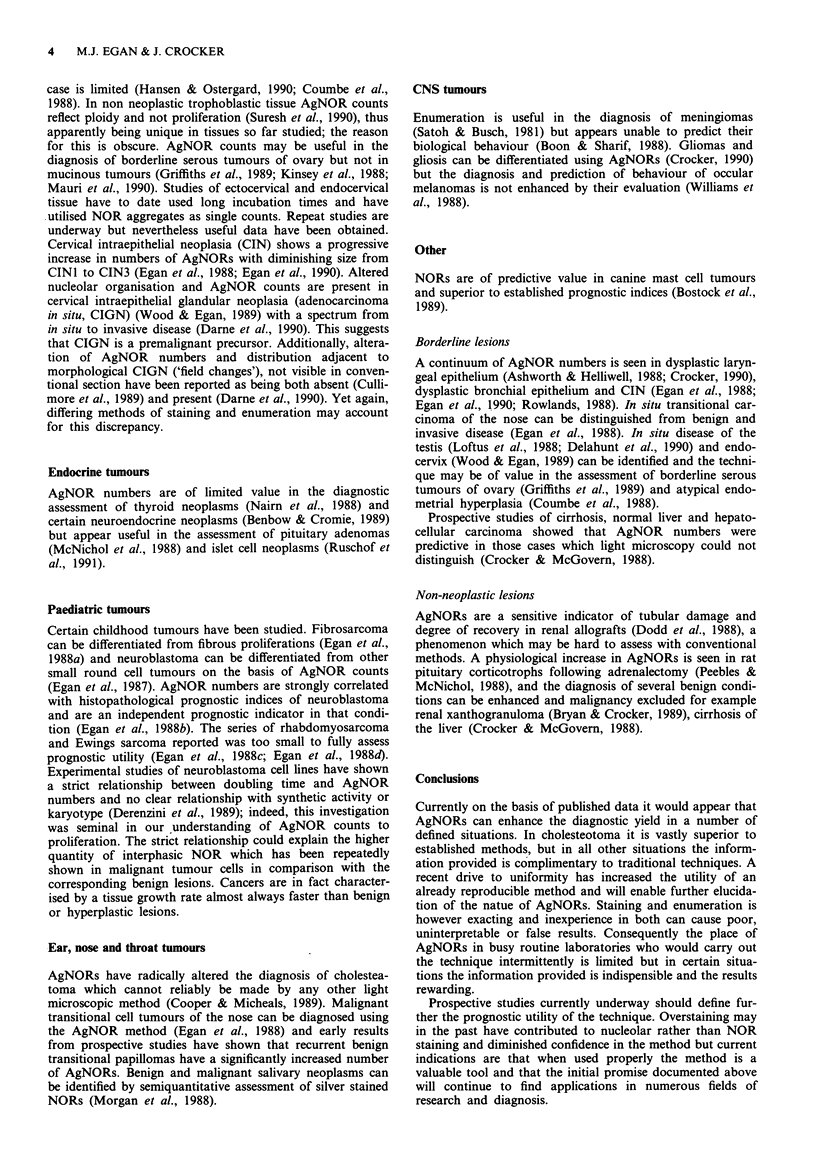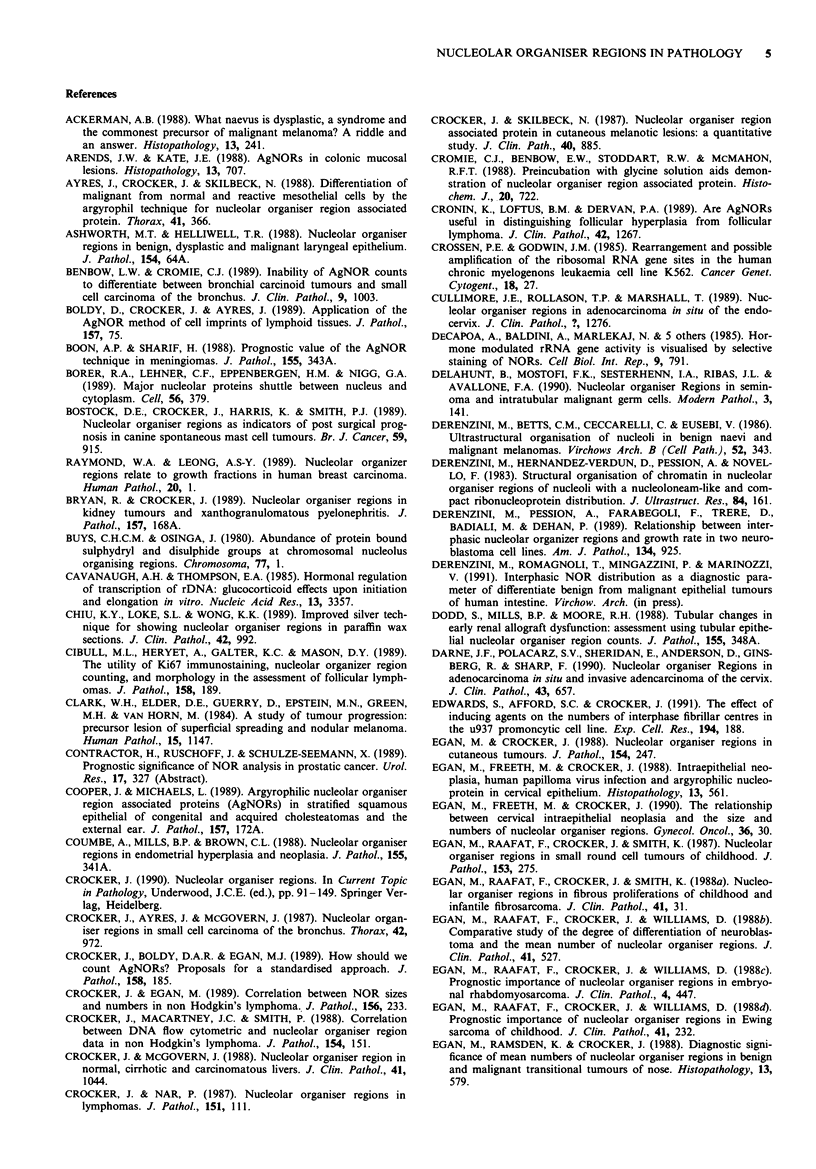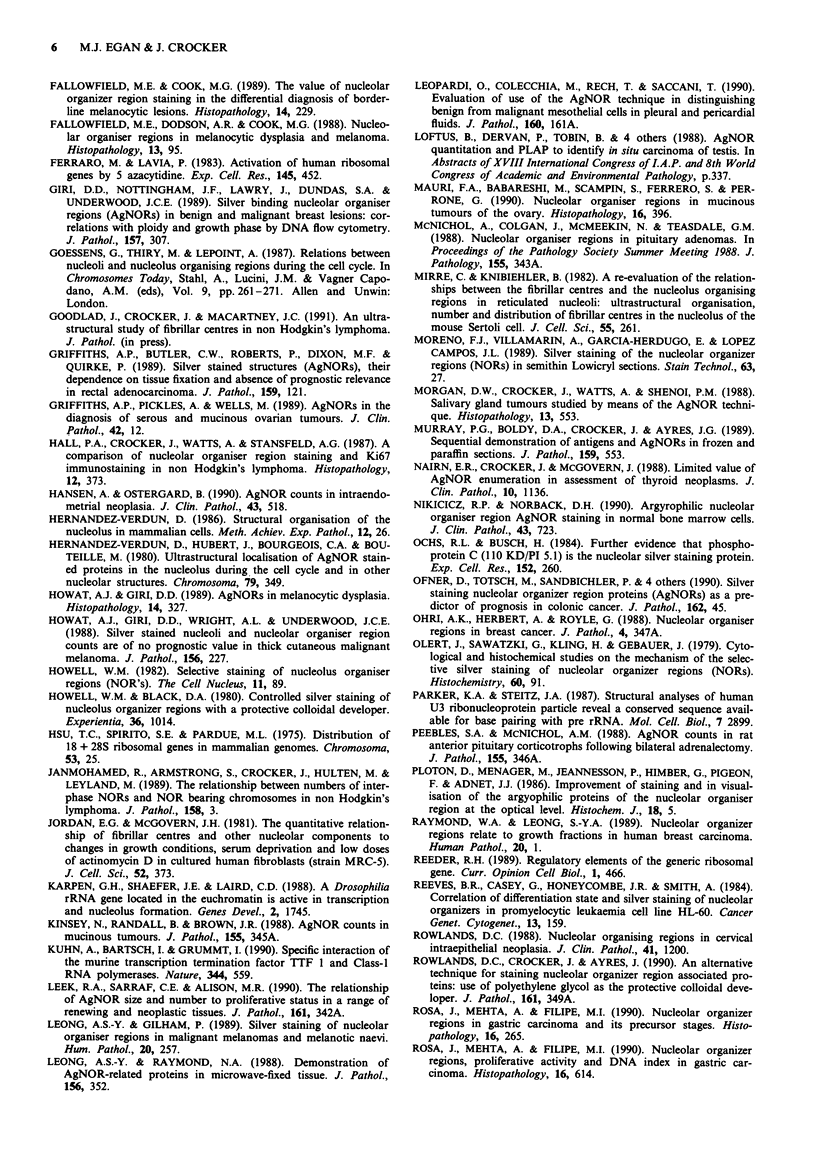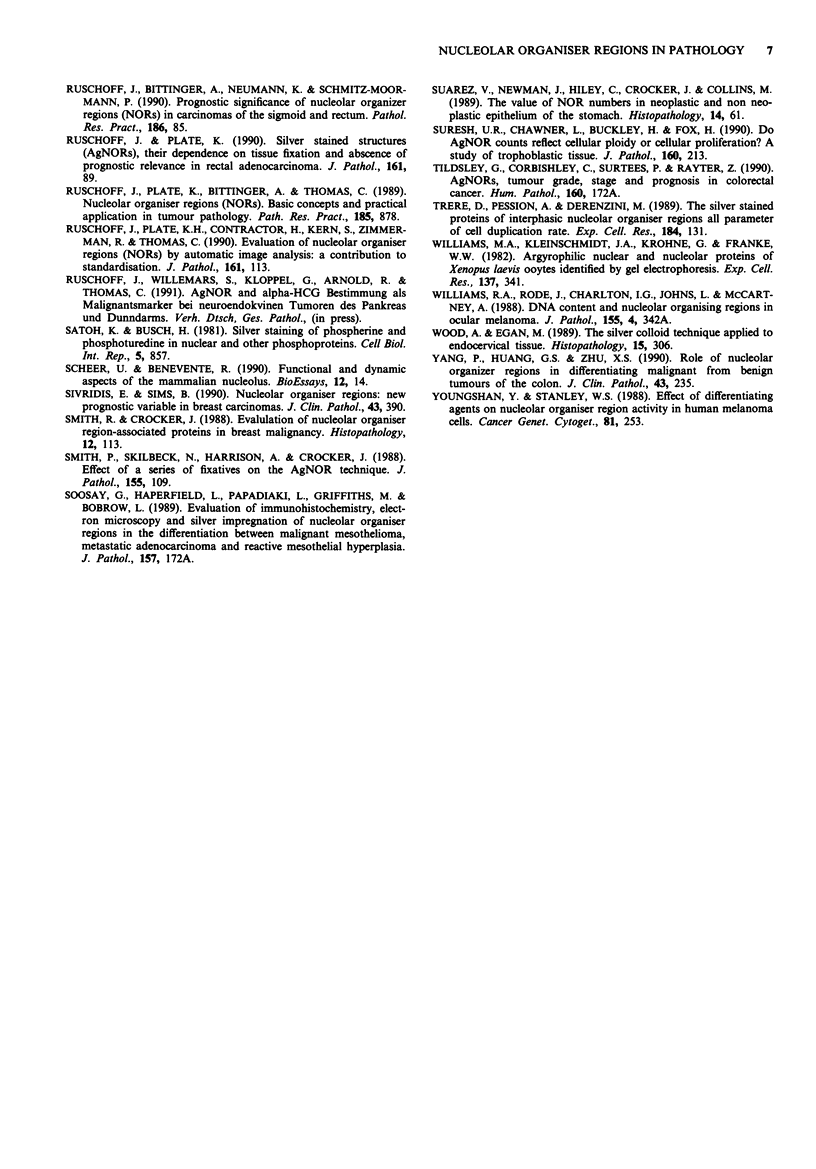# Nucleolar organiser regions in pathology.

**DOI:** 10.1038/bjc.1992.1

**Published:** 1992-01

**Authors:** M. J. Egan, J. Crocker

**Affiliations:** Department of Histopathology, Queen Elizabeth Hospital, Birmingham, UK.


Br. J. Cancer (1992), 65, 1–7

© Macmillan Press Ltd., 1992

**REVIEW**

### Nucleolar organiser regions in pathology

M.J. Egan[1] & J. Crocker[2]

[1]Department of Histopathology, Queen Elizabeth Hospital, Gateshead NE9 6SX; [2]Department of Histopathology, East Birmingham Hospital, Birmingham B9 5ST, UK.

Traditional approaches to pathological diagnosis include macroscopic examination and formalin fixation with the preparation of paraffin sections stained with a variety of histochemical techniques. These routine approaches however may be disappointing in the assessment of many conditions and occasionally fail to render a precise diagnosis or provide adequate prognostic information.

Consequently there has been growing interest within the histopathological community in the state of the nucleus, DNA and proliferation markers. One of the most recent produces of this interst is the study of Nucleolar Organiser Regions (NORs) using a simple silver reduction technique (AgNOR method).

Nucleolar organiser regions are an essential part of the machinery of the nucleolus (Howell, 1982; Derenzini *et al.*, 1983). They can be seen as electron lucent areas at the ultrastructural level (fibrillar centres) which are the interphase equivalent of the condensed chromosomal NORs (Jordan & McGovern, 1981; Hernandez-Verdun, 1986). These structures can be seen at the light microscope level using a variety of techniques including the AgNOR method (Hsu *et al.*, 1975; Ploton *et al.*, 1986). They are involved in ribosome production and potentially qualitative or quantitative changes in interphase NORs may be visible in relation to proliferative activity or transformation and could aid diagnosis or prognostication.

### Physiology and theoretical basis

Mammalian nucleoli have three substructures named according to their ultrastructural appearances (Scheer & Benevente, 1990). These comprise (1) the fibrillar centre, (2) the dense fibrillar component, (3) the granular component. The fibrillar centre is probably the site where the primary r-RNA transcript is generated and contains ribosomal DNA, RNA polymerase I and topoisomerase I(a). It is the equivalent of the interphase NOR seen at the light microscopic level. The dense fibrillar component is the proposed site of early processing of the rRNA precursor and stains with antibodies to fibrillarin, a protein associated with the U3 small nuclear ribonucleoprotein (SnRNP) (Scheer & Benevente, 1990). The granular component is composed of ribosome precursor particles (Scheer & Benevente, 1990). Experiments in *Drosophila* (Karpen *et al.*, 1988) have shown that the nucleolus can form anywhere an active ribosomal gene is located and the central element upon which nucleolar structures assemble is the tandemly-repeated ribosomal DNA that codes for the large rRNA precursor. Evidence is accumulating that vertebrates and possibly many other eukaryotes have a common arrangement of regulatory elements that are arranged as two transcription units per tandem repeat unit (Reeder, 1989). Both of these are transcribed by RNA polymerase I. The second polymerase I transcription unit is thought to influence enhancer function (Reeder, 1989). RNA polymerase I transcription is regulated by at least three polypeptide *trans*-acting factors: UBF and a member of the SL1 class which probably act as components of a step where polymerase recognises a stable complex and initiates transcription, and an initiation factor that associates tightly with the polymerase but which can be separated from it (Cavanaugh & Thompson, 1985). Termination of polymerase I transcription requires specific interaction between the polymerase and a terminator protein (Kuhn *et al.*, 1990). The large RNAs of ribosomes are initially transcribed as single precursor molecules and then processed in a complex series of steps to the mature 18S, 58S and 28S RNAs of functional and ribosomes. This process appears to be directed by SnRNPs. The most abundant nucleolar SnRNP contains RNA, U3, fibrillarin (B36) and at least other six proteins (Parker & Steitz, 1987).

As the rRNA is transcribed and processed it assembles with a large number of specific proteins, the function of most of which is structural. Other proteins do not finally appear in mature ribosomes and have roles in maturation, packaging and transport. One such protein is nucleolin (C23 protein) which is localised in the fibrillar centre and dense fibrillar component. Nucleolin binds to nucleic acids in general, shuttles between cytoplasm and nucleus and has been implicated in the regulation of RNA polymerase I (Borer & Lehner, 1989). Other proteins such as NO38 (B23 protein, numatrin) are also associated with the fibrillar centre (NOR) and may have similar transport functions.

NORs vary in size and shape according to nucleolar transcription. They are intimately related to the cell cycle (Goessens *et al.*, 1987), and may be related to proliferation or ploidy in some circumstances (Suresh *et al.*, 1990; Derenzini *et al.*, 1989; Leek *et al.*, 1990). During prophase the components of the fibrillar centre disperse and in metaphase these structures exist in constant positions on the short arms of the five acrocentric chromosomes (namely 13, 14, 15, 21 and 22). In telophase, tiny granules associate with these NOR bearing chromosomes and these are ultimately rearranged into nucleolar structures. The precise arrangement of chromosomes within these nucleolar structures is not known but it is evident that no clear relationship exists between ploidy and the numbers of NORs at the light and electron microscope levels (Mirre & Knibiehler, 1982).

Since NORs occur in pairs (one on each daughter chromatid) on the five acrocentric chromosomes potentially 20 could be seen at metaphase. However this is a very transient phenomenon and ten can generally be regarded as a full complement in diploid cells. Furthermore the acrocentric chromosomes tend to associate through the satellite regions and this tendancy has a major influence on the number of NORs/fibrillar centres observed. Rearrangements and amplification of the NOR sites have been demonstrated in certain cell lines (Crossen & Godwin, 1985), and regulatory effects of hormones on transcription have been observed (DeCapoa *et*

Received 28 January 1991; and in revised form 10 July 1991.

*al.*, 1985). There is good evidence that differentiation *per se* affects the numbers of NORs observed (Reeves *et al.*, 1984; Edward *et al.*, 1991) but in general the level of cell proliferation as measured by several methods, including DNA flow, cytometry and immunocytochemistry appears to have the major influence (Derenzine *et al.*, 1989; Trere *et al.*, 1989). Ploidy, as assessed by DNA flow cytometry or karyotypic analysis, has little or no effect on interphase NOR scores.

It is clear therefore that ribosomal RNA production is complex and requires numerous enzymes, transcription factors, nuclear RNA, and regulatory factors. These combine to form substructures visible within the nucleolus. The sites of ribosomal transcription can be visualised by a variety of techniques directed against the associated proteins (NORAPS) (Table I). These latter are related specifically to the transcriptionally active sites (Ferraro & Lavia, 1983), although the true NOR, that is, rDNA, was originally identified using radiolabelled rRNA. Some NORAPs notably nucleolin have been shown to stain preferentially with silver as a result of their high electron charge density (Williams *et al.*, 1982), and by virtue of their phosphate (Satoh & Busch, 1981) carboxyl (Olert *et al.*, 1979) and -SH and -s-s-sulphur moeities (Buys & Osinga, 1980).

The silver reduction technique described below has found acceptance as a simple and precise method of NOR demonstration ideally suited to the investigation of NORs at both light and electron microscope levels.

### *The argyrophil (AgNOR) method*

In brief, the one step method consists of mixing silver nitrate and formic acid in optimal proportions with gelatin as a colloid stabliser. Paraffin or frozen sections are incubated in this mixture for variable periods (up to 1 h) and then washed and mounted (Ploton *et al.*, 1986). Ultrastructural and light microscope studies have indicated that this method is remarkably specific as a means for the detection of metaphase and interphase NORs by virtue of their associated proteins. Indeed, sequential staining with radiolabelled rRNA and rDNA has shown correspondence between the binding sites and silver stained NORs on chromosomes and in interphase nucleoli (Hsu *et al.*, 1975).

The silver reaction product is seen as discrete black dots at the light microscope level and these can be enumerated using a × 100 oil immersion lens. Counts in 100 cells are usually made and the results expressed as the mean number of NORs per nucleus. The counts are, of course, not absolute since the NORs themselves are small compared with section thickness. Staining may have to be adjusted slightly to the individual silver binding characteristics of each tissue block and internal controls such as lymphocytes (which have one interphase NOR) are usually employed. With minor modification the technique can be used with success with automatic and semi-automatic image analysis hardware, the total amount of argyrophil material per nucleus being measured, rather than the number of sites counted (Ruschoff *et al.*, 1989; Ruschoff *et al.*, 1990). The one step method (Howell & Black, 1980), is an ingenious modification of the more time consuming original three step technique and has itself undergone modification including preincubation with glycine to reduce incubation time (Cromie *et al.*, 1988), the use of polyethylene glyco as a protective colloidal developer (in place of gelatin), to reduce non-specific deposit (Rowlands *et al.*, 1990), and the inclusion of a celloidin film, also to reduce nonspecific deposit (Chiu *et al.*, 1989). Furthermore, minor adaptations have been employed to allow application to cell imprints (Boldy *et al.*, 1989; Ruschoff *et al.*, 1989), cytospin preparations (Ruschoff *et al.*, 1989), and semithin methacrylate sections (Moreno *et al.*, 1989).

**Table I** Techniques for demonstration of NORs

| *Reagent* | *Target* |
|---|---|
| Radiolabelled rRNA | rDNA |
| Silver colloid (AgNOR) | NORAPs |
| Mercuridibromfluorescein | NORAPs |
| Bismuth ions | 100k NORAP |
| Antibodies | Various NORAP epitopes |

### *Technical aspects*

The one step silver method appears to give reproducible and specific staining results (Hernandez-Verdun *et al.*, 1980; Buys & Osinga, 1980; Ochs & Busch, 1984). From the early stages however, it was clear that uniformity with respect to fixation, technique and enumeration were required to optimise consistent discrimination of silver stained NORs (AgNORs).

In general, prompt fixation in 10% formol saline is perfectly satisfactory (Crocker *et al.*, 1989; Smith *et al.*, 1988). Alcoholic fixatives may give stronger staining, but picric acid- and mercury-containing fixatives are highly deleterious (Smith *et al.*, 1988). The technique is readily applicable to microwave fixed tissue (Leong & Raymond, 1988), and frozen sections (Murray *et al.*, 1989), furthermore sequential staining with immunohistochemical reagents has no adverse effects (Murray *et al.*, 1989). The latter may be most useful where neoplasms are highly heterogeneous, the immunohistochemical method being used to pinpoint (or exclude) the cells in which AgNOR enumeration is required.

Early studies counted all separate silver stained structures after a constant incubation time but it is now clear that minor alterations according to internal controls are advisable to allow counting of subsiduary dots within nucleolar associations (Crocker *et al.*, 1989; Ruschoff & Plate, 1990). This recent drive to uniformity has obviated certain early work and further studies are currently underway.

### *Application*

The potential of the one step silver colloid technique is histopathology was first investigated by Ploton *et al.* (1986) who stained an unspecified number of specimens of prostate and concluded that the method might prove useful in tumour diagnosis.

Numerous studies have subsequently appeared in the literature investigating malignancy, borderline malignancy and a variety of non neoplastic conditions. These are discussed below and conditions in which AgNORs may be of diagnostic or prognostic use are summarised in Table II.

### Malignant conditions – lymphoma

The potential of AgNOR identification and quantification has been investigated in the greatest detail in lymphomas. The assessment and grading of non Hodgkin's lymphoma (NHL) is a clinically crucial procedure and accurate evalua-

**Table IIa** Situations in which histological evaluation of AgNORs provide information additional to traditional histopathological methods

| | |
|---|---|
| Lymphoma grading | Islet cell neoplasms |
| Pleural malignancy | Small cell tumours of childhood |
| Small cell carcinoma of lung | Infantile fibrosarcoma |
| Cholesteatoma | Gliomas |
| *In situ* carcinoma of the testis | Hepatocellular carcinomas |
| Borderline serous tumours of ovary | Rejection of renal allografts |

**Table IIB** Conditions in which histological evaluation of AgNORs appear to be of prognostic value

| | |
|---|---|
| Colonic neoplasia | Neuroblastoma |
| Gastric cancer | Mast cell tumours |
| ? Breast cancer | |
| Prostatic cancer | |

tion currently requires a battery of special procedures including light and electron microscopy, immunohistochemistry, histochemistry and DNA technology. It would not appear that NOR enumeration is of value in the study of NHL in both sections (Crocker & Nar, 1987), and cell imprints (Boldy *et al.*, 1989) and also in the related field of bone marrow aspirate evaluation (Nikicicz & Norback, 1990).

Initial studies have indicated that enumeration (Boldy *et al.*, 1989) or even morphometric analysis of the size of fibrillar centres (Crocker & Egan, 1989; Goodlad *et al.*, 1991) can help in the identification of high from low grade types. Two further studies of follicular lymphoma have shown that when used alone AgNORs cannot distinguish follicular hyperplasia from this distinct subtype of low grade lymphoma (Cronin *et al.*, 1989; Cibull *et al.*, 1989).

Subsequent studies have shown that the number of AgNORS observed are closely related to the percentage of cells recognised by the antibody Ki 67 (Hall *et al.*, 1987). This antibody binds to a nuclear antigen expressed in all cell cycle phases except G. Furthermore, in lymphomas, AgNOR numbers show no relationship to ploidy as assessed by DNA flow cytometry (Crocker *et al.*, 1988), or numbers of AgNORs or chromosomes themselves observed in metaphase spreads (Janmohamed *et al.*, 1989), but have a close correlation with the percentage S phase cells (Crocker *et al.*, 1988) as determined by DNA flow cytometry. It is likely therefore that sizes and numbers of AgNORs are dependent on the proliferative status of the cell or tissue studied.

### Gastrointestinal tract

Several studies of rectal and colonic neoplasia have failed to demonstrate the value of evaluation of AgNORs with regard to clinical outcome (Griffiths *et al.*, 1989; Arends & Kate, 1988; Tildsley *et al.*, 1990). Other studies have discriminated accurately between benign and malignant conditions and demonstrated prognostic significance (Ruschoff & Plate, 1990; Young *et al.*, 1990; Ofner *et al.*, 1990; Ruschoff *et al.*, 1990; Derenzini *et al.*, 1991). This apparent contrast may be explicable by incubation time alone. In the former studies, long incubation times may have caused overstaining and led to false fusion of AgNORs lending to decreased scores. It would therefore appear that athough AgNORs have no real advantage over established techniques in the diagnosis of colonic cancer, when used in the assessment of prognosis they may have considerable utility.

Using AgNOR scores as markers of proliferative activity in gastric epithelium Rosa *et al.* (Mehta & Filipe, 1990*a*) have demonstrated significant differences between dysplastic, malignant and normal epithelium. As in lymphoma this group subsequently showed no relationship between AgNOR counts and DNA index (Rosa *et al.*, 1990*b*; Ruschoff & Plate, 1990). Ruschoff *et al.* demonstrated a prognostic significance in gastric carcinoma. Early studies using longer but variable incubation times showed overlap between benign, regenerative and malignant epithelium (Suarez *et al.*, 1989).

### Respiratory tract

Studies of pulmonary tumours have also yielded conflicting results, once again explicable on the basis of the technique employed. We have found AgNOR enumeration to be useful in the evaluation of malignancy in pleural aspirates, pleural biopsies and follow-up post mortem tissue (unpublished observations). The method distinguishes reliably between malignancy and hyperplasia but cannot differentiate mesothelioma from adenocarcinoma (Ayres *et al.*, 1988). Other works have found it to be of limited use in this context (Soosay *et al.*, 1989; Leopardi *et al.*, 1990), but in the opinion of the authors NOR staining enhances the diagnostic yield, and provides information additional to that derived from traditional methods. The method can distinguish small cell carcinoma of bronchus from lymphocytes which is particularly useful in small biopsies (Crocker *et al.*, 1987), but cannot distinguish carcinoids, atypical carcinoids (Benbow & Cromie, 1989) or low grade NHL (Crocker *et al.*, 1987).

In other situations the use of AgNOR counts is yet to be proven. Counts in squamous carcinomas of larynx and bronchus are related inversely to differentiation (Ashworth & Helliwell, 1988; Crocker, 1990); however, squamous tumours of pharynx and larynx cannot be separated on the basis of AgNOR counts (Bryan *et al.*, 1990). It is not yet certain whether AgNORs are related to prognosis for the different histological varieties of lung cancer and investigation are currently underway. Studies of AgNORs and therapeutic response are needed, not only in lung tumours but in many other contexts.

### Breast

In the case of breast disease diagnostic AgNOR studies have been published which show enumeration to be useful (Smith & Crocker, 1988; Ohri *et al.*, 1988; Raymond & Leong, 1989) or of limited value (Giri *et al.*, 1989). Prognostic studies have indicated that counting and AgNOR morphology may provide information with regard to nodal metastases, supplementary to that obtained by established methodology (Sividis & Sims, 1990). Currently there is no indication that AgNOR enumeration will prove to be of value in the histological diagnosis of breast lesions, but potentially useful applications are to cytologically suspect, and equivocal fine needle aspirate specimens and to prognosis.

### Skin

The method is applicable to skin pathology although it does not appear superior to established methodology. Adnexal tumours and epidermal tumours can be identified on the basis of AgNORs and the spectrum of differentiation of squamous carcinoma is reflected by a range of AgNOR numbers (Egan & Crocker, 1988). Studies of melanocytic tumours are hampered by the argyrophilia of melanin (Crocker & Skilbeck, 1987); nevertheless it has been shown that AgNOR staining is useful in separating melanocytic naevi from malignant melanomas (Crocker & Skilbeck, 1987; Leong & Gilham, 1989; Derenzini *et al.*, 1986) but of limited value as a prognostic indicator (Howat *et al.*, 1988). The value of NOR staining in borderline lesions is currently uncertain (Fallowfield *et al.*, 1988; Fallowfield & Cook, 1989; Howat & Giri, 1989). Indeed the very existance of dysplastic naevi as a clinical and pathological entity is a matter of considerable debate (Clark *et al.*, 1984; Ackerman, 1988). The only studies of AgNORs in dysplastic naevi suffer from the same overstaining as other earlier AgNOR studies and consequently may have a limited contribution (Fallowfield *et al.*, 1988; Fallowfield & Cook, 1989). Differentiation of melanoma cell lines under the influence of retinoic acid is associated with a reversible decrease in the number of AgNORs (Youngshan & Stanley, 1988). No clinical chemotherapeutic response studies are available.

### Genitourinary system

The diagnosis and grading of renal carcinoma using evaluation of AgNORs by light microscopy or image analysis has been shown to be of great value and readily applicable to cytological specimens (Ruschoff *et al.*, 1989). The diagnosis of *in situ* carcinoma of the testis can be made using AgNOR counts (Loftus *et al.*, 1988) and spermatic seminoma and typical seminoma showed different distributions of AgNORs (Delahunt *et al.*, 1990). AgNOR analysis in prostatic adenocarcinomas has considerable prognostic utility (Contractor *et al.*, 1989). Significant differences of AgNOR counts are seen for pathological endometrium and neoplastic endometrium but since absolute differences are small the use in any single case is limited (Hansen & Ostergard, 1990; Coumbe *et al.*, 1988). In non neoplastic trophoblastic tissue AgNOR counts reflect ploidy and not proliferation (Suresh *et al.*, 1990), thus apparently being unique in tissues so far studied; the reason for this is obscure. AgNOR counts may be useful in the diagnosis of borderline serous tumours of ovary but not in mucinous tumours (Griffiths *et al.*, 1989; Kinsey *et al.*, 1988; Mauri *et al.*, 1990). Studies of ectocervical and endocervical tissue have to date used long incubation times and have utilised NOR aggregates as single counts. Repeat studies are underway but nevertheless useful data have been obtained. Cervical intraepithelial neoplasia (CIN) shows a progressive increase in numbers of AgNORs with diminishing size from CIN1 to CIN3 (Egan *et al.*, 1988; Egan *et al.*, 1990). Altered nucleolar organisation and AgNOR counts are present in cervical intraepithelial glandular neoplasia (adenocarcinoma *in situ*, CIGN) (Wood & Egan, 1989) with a spectrum from *in situ* to invasive disease (Darne *et al.*, 1990). This suggests that CIGN is a premalignant precursor. Additionally, alteration of AgNOR numbers and distribution adjacent to morphological CIGN ('field changes'), not visible in conventional section have been reported as being both absent (Cullimore *et al.*, 1989) and present (Darne *et al.*, 1990). Yet again, differing methods of staining and enumeration may account for this discrepancy.

### Endocrine tumours

AgNOR numbers are of limited value in the diagnostic assessment of thyroid neoplasms (Nairn *et al.*, 1988) and certain neuroendocrine neoplasms (Benbow & Cromie, 1989) but appear useful in the assessment of pituitary adenomas (McNichol *et al.*, 1988) and islet cell neoplasms (Ruschof *et al.*, 1991).

### Paediatric tumours

Certain childhood tumours have been studied. Fibrosarcoma can be differentiated from fibrous proliferations (Egan *et al.*, 1988*a*) and neuroblastoma can be differentiated from other small round cell tumours on the basis of AgNOR counts (Egan *et al.*, 1987). AgNOR numbers are strongly correlated with histopathological prognostic indices of neuroblastoma and are an independent prognostic indicator in that condition (Egan *et al.*, 1988*b*). The series of rhabdomyosarcoma and Ewings sarcoma reported was too small to fully assess prognostic utility (Egan *et al.*, 1988*c*; Egan *et al.*, 1988*d*). Experimental studies of neuroblastoma cell lines have shown a strict relationship between doubling time and AgNOR numbers and no clear relationship with synthetic activity or karyotype (Derenzini *et al.*, 1989); indeed, this investigation was seminal in our understanding of AgNOR counts to proliferation. The strict relationship could explain the higher quantity of interphasic NOR which has been repeatedly shown in malignant tumour cells in comparison with the corresponding benign lesions. Cancers are in fact characterised by a tissue growth rate almost always faster than benign or hyperplastic lesions.

### Ear, nose and throat tumours

AgNORs have radically altered the diagnosis of cholesteatoma which cannot reliably be made by any other light microscopic method (Cooper & Micheals, 1989). Malignant transitional cell tumours of the nose can be diagnosed using the AgNOR method (Egan *et al.*, 1988) and early results from prospective studies have shown that recurrent benign transitional papillomas have a significantly increased number of AgNORs. Benign and malignant salivary neoplasms can be identified by semiquantitative assessment of silver stained NORs (Morgan *et al.*, 1988).

### CNS tumours

Enumeration is useful in the diagnosis of meningiomas (Satoh & Busch, 1981) but appears unable to predict their biological behaviour (Boon & Sharif, 1988). Gliomas and gliosis can be differentiated using AgNORs (Crocker, 1990) but the diagnosis and prediction of behaviour of occular melanomas is not enhanced by their evaluation (Williams *et al.*, 1988).

### Other

NORs are of predictive value in canine mast cell tumours and superior to established prognostic indices (Bostock *et al.*, 1989).

### *Borderline lesions*

A continuum of AgNOR numbers is seen in dysplastic laryngeal epithelium (Ashworth & Helliwell, 1988; Crocker, 1990), dysplastic bronchial epithelium and CIN (Egan *et al.*, 1988; Egan *et al.*, 1990; Rowlands, 1988). *In situ* transitional carcinoma of the nose can be distinguished from benign and invasive disease (Egan *et al.*, 1988). *In situ* disease of the testis (Loftus *et al.*, 1988; Delahunt *et al.*, 1990) and endocervix (Wood & Egan, 1989) can be identified and the technique may be of value in the assessment of borderline serous tumours of ovary (Griffiths *et al.*, 1989) and atypical endometrial hyperplasia (Coumbe *et al.*, 1988).

Prospective studies of cirrhosis, normal liver and hepatocellular carcinoma showed that AgNOR numbers were predictive in those cases which light microscopy could not distinguish (Crocker & McGovern, 1988).

### *Non-neoplastic lesions*

AgNORs are a sensitive indicator of tubular damage and degree of recovery in renal allografts (Dodd *et al.*, 1988), a phenomenon which may be hard to assess with conventional methods. A physiological increase in AgNORs is seen in rat pituitary corticotrophs following adrenalectomy (Peebles & McNichol, 1988), and the diagnosis of several benign conditions can be enhanced and malignancy excluded for example renal xanthogranuloma (Bryan & Crocker, 1989), cirrhosis of the liver (Crocker & McGovern, 1988).

### Conclusions

Currently on the basis of published data it would appear that AgNORs can enhance the diagnostic yield in a number of defined situations. In cholesteotoma it is vastly superior to established methods, but in all other situations the information provided is complimentary to traditional techniques. A recent drive to uniformity has increased the utility of an already reproducible method and will enable further elucidation of the natue of AgNORs. Staining and enumeration is however exacting and inexperience in both can cause poor, uninterpretable or false results. Consequently the place of AgNORs in busy routine laboratories who would carry out the technique intermittently is limited but in certain situations the information provided is indispensible and the results rewarding.

Prospective studies currently underway should define further the prognostic utility of the technique. Overstaining may in the past have contributed to nucleolar rather than NOR staining and diminished confidence in the method but current indications are that when used properly the method is a valuable tool and that the initial promise documented above will continue to find applications in numerous fields of research and diagnosis.

### References

ACKERMAN, A.B. (1988). What naevus is dysplastic, a syndrome and the commonest precursor of malignant melanoma? A riddle and an answer. *Histopathology*, **13**, 241.

ARENDS, J.W. & KATE, J.E. (1988). AgNORs in colonic mucosal lesions. *Histopathology*, **13**, 707.

AYRES, J., CROCKER, J. & SKILBECK, N. (1988). Differentiation of malignant from normal and reactive mesothelial cells by the argyrophil technique for nucleolar organiser region associated protein. *Thorax*, **41**, 366.

ASHWORTH, M.T. & HELLIWELL, T.R. (1988). Nucleolar organiser regions in benign, dysplastic and malignant laryngeal epithelium. *J. Pathol.*, **154**, 64A.

BENBOW, L.W. & CROMIE, C.J. (1989). Inability of AgNOR counts to differentiate between bronchial carcinoid tumours and small cell carcinoma of the bronchus. *J. Clin. Pathol.*, **9**, 1003.

BOLDY, D., CROCKER, J. & AYRES, J. (1989). Application of the AgNOR method of cell imprints of lymphoid tissues. *J. Pathol.*, **157**, 75.

BOON, A.P. & SHARIF, H. (1988). Prognostic value of the AgNOR technique in meningiomas. *J. Pathol.*, **155**, 343A.

BORER, R.A., LEHNER, C.F., EPPENBERGEN, H.M. & NIGG, G.A. (1989). Major nucleolar proteins shuttle between nucleus and cytoplasm. *Cell*, **56**, 379.

BOSTOCK, D.E., CROCKER, J., HARRIS, K. & SMITH, P.J. (1989). Nucleolar organiser regions as indicators of post surgical prognosis in canine spontaneous mast cell tumours. *Br. J. Cancer*, **59**, 915.

RAYMOND, W.A. & LEONG, A.S-Y. (1989). Nucleolar organizer regions relate to growth fractions in human breast carcinoma. *Human Pathol.*, **20**, 1.

BRYAN, R. & CROCKER, J. (1989). Nucleolar organiser regions in kidney tumours and xanthogranulomatous pyelonephritis. *J. Pathol.*, **157**, 168A.

BUYS, C.H.C.M. & OSINGA, J. (1980). Abundance of protein bound sulphydryl and disulphide groups at chromosomal nucleolus organising regions. *Chromosoma*, **77**, 1.

CAVANAUGH, A.H. & THOMPSON, E.A. (1985). Hormonal regulation of transcription of rDNA: glucocorticoid effects upon initiation and elongation *in vitro*. *Nucleic Acid Res.*, **13**, 3357.

CHIU, K.Y., LOKE, S.L. & WONG, K.K. (1989). Improved silver technique for showing nucleolar organiser regions in paraffin wax sections. *J. Clin. Pathol.*, **42**, 992.

CIBULL, M.L., HERYET, A., GALTER, K.C. & MASON, D.Y. (1989). The utility of Ki67 immunostaining, nucleolar organizer region counting, and morphology in the assessment of follicular lymphomas. *J. Pathol.*, **158**, 189.

CLARK, W.H., ELDER, D.E., GUERRY, D., EPSTEIN, M.N., GREEN, M.H. & VAN HORN, M. (1984). A study of tumour progression: precursor lesion of superficial spreading and nodular melanoma. *Human Pathol.*, **15**, 1147.

CONTRACTOR, H., RUSCHOFF, J. & SCHULZE-SEEMANN, X. (1989). Prognostic significance of NOR analysis in prostatic cancer. *Urol. Res.*, **17**, 327 (Abstract).

COOPER, J. & MICHAELS, L. (1989). Argyrophilic nucleolar organiser region associated proteins (AgNORs) in stratified squamous epithelial of congenital and acquired cholesteatomas and the external ear. *J. Pathol.*, **157**, 172A.

COUMBE, A., MILLS, B.P. & BROWN, C.L. (1988). Nucleolar organiser regions in endometrial hyperplasia and neoplasia. *J. Pathol.*, **155**, 341A.

CROCKER, J. (1990). Nucleolar organiser regions. In *Current Topic in Pathology*, Underwood, J.C.E. (ed.), pp. 91–149. Springer Verlag, Heidelberg.

CROCKER, J., AYRES, J. & MCGOVERN, J. (1987). Nucleolar organiser regions in small cell carcinoma of the bronchus. *Thorax*, **42**, 972.

CROCKER, J., BOLDY, D.A.R. & EGAN, M.J. (1989). How should we count AgNORs? Proposals for a standardised approach. *J. Pathol.*, **158**, 185.

CROCKER, J. & EGAN, M. (1989). Correlation between NOR sizes and numbers in non Hodgkin's lymphoma. *J. Pathol.*, **156**, 233.

CROCKER, J., MACARTNEY, J.C. & SMITH, P. (1988). Correlation between DNA flow cytometric and nucleolar organiser region data in non Hodgkin's lymphoma. *J. Pathol.*, **154**, 151.

CROCKER, J. & MCGOVERN, J. (1988). Nucleolar organiser region in normal, cirrhotic and carcinomatous livers. *J. Clin. Pathol.*, **41**, 1044.

CROCKER, J. & NAR, P. (1987). Nucleolar organiser regions in lymphomas. *J. Pathol.*, **151**, 111.

CROCKER, J. & SKILBECK, N. (1987). Nucleolar organiser region associated protein in cutaneous melanotic lesions: a quantitative study. *J. Clin. Path.*, **40**, 885.

CROMIE, C.J., BENBOW, E.W., STODDART, R.W. & MCMAHON, R.F.T. (1988). Preincubation with glycine solution aids demonstration of nucleolar organiser region associated protein. *Histochem. J.*, **20**, 722.

CRONIN, K., LOFTUS, B.M. & DERVAN, P.A. (1989). Are AgNORs useful in distinguishing follicular hyperplasia from follicular lymphoma. *J. Clin. Pathol.*, **42**, 1267.

CROSSEN, P.E. & GODWIN, J.M. (1985). Rearrangement and possible amplification of the ribosomal RNA gene sites in the human chronic myelogenons leukaemia cell line K562. *Cancer Genet. Cytogent.*, **18**, 27.

CULLIMORE, J.E., ROLLASON, T.P. & MARSHALL, T. (1989). Nucleolar organiser regions in adenocarcinoma *in situ* of the endocervix. *J. Clin. Pathol.*, **?**, 1276.

DECAPOA, A., BALDINI, A., MARLEKAJ, N. & 5 others (1985). Hormone modulated rRNA gene activity is visualised by selective staining of NORs. *Cell Biol. Int. Rep.*, **9**, 791.

DELAHUNT, B., MOSTOFI, F.K., SESTERHENN, I.A., RIBAS, J.L. & AVALLONE, F.A. (1990). Nucleolar organiser Regions in seminoma and intratubular malignant germ cells. *Modern Pathol.*, **3**, 141.

DERENZINI, M., BETTS, C.M., CECCARELLI, C. & EUSEBI, V. (1986). Ultrastructural organisation of nucleoli in benign naevi and malignant melanomas. *Virchows Arch. B (Cell Path.)*, **52**, 343.

DERENZINI, M., HERNANDEZ-VERDUN, D., PESSION, A. & NOVELLO, F. (1983). Structural organisation of chromatin in nucleolar organiser regions of nucleoli with a nucleoloneam-like and compact ribonucleoprotein distribution. *J. Ultrastruct. Res.*, **84**, 161.

DERENZINI, M., PESSION, A., FARABEGOLI, F., TRERE, D., BADIALI, M. & DEHAN, P. (1989). Relationship between interphasic nucleolar organizer regions and growth rate in two neuroblastoma cell lines. *Am. J. Pathol.*, **134**, 925.

DERENZINI, M., ROMAGNOLI, T., MINGAZZINI, P. & MARINOZZI, V. (1991). Interphasic NOR distribution as a diagnostic parameter of differentiate benign from malignant epithelial tumours of human intestine. *Virchow. Arch.* (in press).

DODD, S., MILLS, B.P. & MOORE, R.H. (1988). Tubular changes in early renal allograft dysfunction: assessment using tubular epithelial nucleolar organiser region counts. *J. Pathol.*, **155**, 348A.

DARNE, J.F., POLACARZ, S.V., SHERIDAN, E., ANDERSON, D., GINSBERG, R. & SHARP, F. (1990). Nucleolar organiser Regions in adenocarcinoma *in situ* and invasive adencarcinoma of the cervix. *J. Clin. Pathol.*, **43**, 657.

EDWARDS, S., AFFORD, S.C. & CROCKER, J. (1991). The effect of inducing agents on the numbers of interphase fibrillar centres in the u937 promoncytic cell line. *Exp. Cell. Res.*, **194**, 188.

EGAN, M. & CROCKER, J. (1988). Nucleolar organiser regions in cutaneous tumours. *J. Pathol.*, **154**, 247.

EGAN, M., FREETH, M. & CROCKER, J. (1988). Intraepithelial neoplasia, human papilloma virus infection and argyrophilic nucleoprotein in cervical epithelium. *Histopathology*, **13**, 561.

EGAN, M., FREETH, M. & CROCKER, J. (1990). The relationship between cervical intraepithelial neoplasia and the size and numbers of nucleolar organiser regions. *Gynecol. Oncol.*, **36**, 30.

EGAN, M., RAAFAT, F., CROCKER, J. & SMITH, K. (1987). Nucleolar organiser regions in small round cell tumours of childhood. *J. Pathol.*, **153**, 275.

EGAN, M., RAAFAT, F., CROCKER, J. & SMITH, K. (1988*a*). Nucleolar organiser regions in fibrous proliferations of childhood and infantile fibrosarcoma. *J. Clin. Pathol.*, **41**, 31.

EGAN, M., RAAFAT, F., CROCKER, J. & WILLIAMS, D. (1988*b*). Comparative study of the degree of differentiation of neuroblastoma and the mean number of nucleolar organiser regions. *J. Clin. Pathol.*, **41**, 527.

EGAN, M., RAAFAT, F., CROCKER, J. & WILLIAMS, D. (1988*c*). Prognostic importance of nucleolar organiser regions in embryonal rhabdomyosarcoma. *J. Clin. Pathol.*, **4**, 447.

EGAN, M., RAAFAT, F., CROCKER, J. & WILLIAMS, D. (1988*d*). Prognostic importance of nucleolar organiser regions in Ewing sarcoma of childhood. *J. Clin. Pathol.*, **41**, 232.

EGAN, M., RAMSDEN, K. & CROCKER, J. (1988). Diagnostic significance of mean numbers of nucleolar organiser regions in benign and malignant transitional tumours of nose. *Histopathology*, **13**, 579.

FALLOWFIELD, M.E. & COOK, M.G. (1989). The value of nucleolar organizer region staining in the differential diagnosis of borderline melanocytic lesions. *Histopathology*, **14**, 229.

FALLOWFIELD, M.E., DODSON, A.R. & COOK, M.G. (1988). Nucleolar organiser regions in melanocytic dysplasia and melanoma. *Histopathology*, **13**, 95.

FERRARO, M. & LAVIA, P. (1983). Activation of human ribosomal genes by 5 azacytidine. *Exp. Cell. Res.*, **145**, 452.

GIRI, D.D., NOTTINGHAM, J.F., LAWRY, J., DUNDAS, S.A. & UNDERWOOD, J.C.E. (1989). Silver binding nucleolar organiser regions (AgNORs) in benign and malignant breast lesions: correlations with ploidy and growth phase by DNA flow cytometry. *J. Pathol.*, **157**, 307.

GOESSENS, G., THIRY, M. & LEPOINT, A. (1987). Relations between nucleoli and nucleolus organising regions during the cell cycle. In *Chromosomes Today*, Stahl, A., Lucini, J.M. & Vagner Capodano, A.M. (eds), Vol. 9, pp. 261–271. Allen and Unwin: London.

GOODLAD, J., CROCKER, J. & MACARTNEY, J.C. (1991). An ultrastructural study of fibrillar centres in non Hodgkin's lymphoma. *J. Pathol.* (in press).

GRIFFITHS, A.P., BUTLER, C.W., ROBERTS, P., DIXON, M.F. & QUIRKE, P. (1989). Silver stained structures (AgNORs), their dependence on tissue fixation and absence of prognostic relevance in rectal adenocarcinoma. *J. Pathol.*, **159**, 121.

GRIFFITHS, A.P., PICKLES, A. & WELLS, M. (1989). AgNORs in the diagnosis of serous and mucinous ovarian tumours. *J. Clin. Pathol.*, **42**, 12.

HALL, P.A., CROCKER, J., WATTS, A. & STANSFELD, A.G. (1987). A comparison of nucleolar organiser region staining and Ki67 immunostaining in non Hodgkin's lymphoma. *Histopathology*, **12**, 373.

HANSEN, A. & OSTERGARD, B. (1990). AgNOR counts in intraendometrial neoplasia. *J. Clin. Pathol.*, **43**, 518.

HERNANDEZ-VERDUN, D. (1986). Structural organisation of the nucleolus in mammalian cells. *Meth. Achiev. Exp. Pathol.*, **12**, 26.

HERNANDEZ-VERDUN, D., HUBERT, J., BOURGEOIS, C.A. & BOUTEILLE, M. (1980). Ultrastructural localisation of AgNOR stained proteins in the nucleolus during the cell cycle and in other nucleolar structures. *Chromosoma*, **79**, 349.

HOWAT, A.J. & GIRI, D.D. (1989). AgNORs in melanocytic dysplasia. *Histopathology*, **14**, 327.

HOWAT, A.J., GIRI, D.D., WRIGHT, A.L. & UNDERWOOD, J.C.E. (1988). Silver stained nucleoli and nucleolar organiser region counts are of no prognostic value in thick cutaneous malignant melanoma. *J. Pathol.*, **156**, 227.

HOWELL, W.M. (1982). Selective staining of nucleolus organiser regions (NOR's). *The Cell Nucleus*, **11**, 89.

HOWELL, W.M. & BLACK, D.A. (1980). Controlled silver staining of nucleolus organizer regions with a protective colloidal developer. *Experientia*, **36**, 1014.

HSU, T.C., SPIRITO, S.E. & PARDUE, M.L. (1975). Distribution of 18 + 28S ribosomal genes in mammalian genomes. *Chromosoma*, **53**, 25.

JANMOHAMED, R., ARMSTRONG, S., CROCKER, J., HULTEN, M. & LEYLAND, M. (1989). The relationship between numbers of interphase NORs and NOR bearing chromosomes in non Hodgkin's lymphoma. *J. Pathol.*, **158**, 3.

JORDAN, E.G. & MCGOVERN, J.H. (1981). The quantitative relationship of fibrillar centres and other nucleolar components to changes in growth conditions, serum deprivation and low doses of actinomycin D in cultured human fibroblasts (strain MRC-5). *J. Cell. Sci.*, **52**, 373.

KARPEN, G.H., SHAEFER, J.E. & LAIRD, C.D. (1988). A *Drosophilia* rRNA gene located in the euchromatin is active in transcription and nucleolus formation. *Genes Devel.*, **2**, 1745.

KINSEY, N., RANDALL, B. & BROWN, J.R. (1988). AgNOR counts in mucinous tumours. *J. Pathol.*, **155**, 345A.

KUHN, A., BARTSCH, I. & GRUMMT, I. (1990). Specific interaction of the murine transcription termination factor TTF 1 and Class-1 RNA polymerases. *Nature*, **344**, 559.

LEEK, R.A., SARRAF, C.E. & ALISON, M.R. (1990). The relationship of AgNOR size and number to proliferative status in a range of renewing and neoplastic tissues. *J. Pathol.*, **161**, 342A.

LEONG, A.S.-Y. & GILHAM, P. (1989). Silver staining of nucleolar organiser regions in malignant melanomas and melanotic naevi. *Hum. Pathol.*, **20**, 257.

LEONG, A.S.-Y. & RAYMOND, N.A. (1988). Demonstration of AgNOR-related proteins in microwave-fixed tissue. *J. Pathol.*, **156**, 352.

LEOPARDI, O., COLECCHIA, M., RECH, T. & SACCANI, T. (1990). Evaluation of use of the AgNOR technique in distinguishing benign from malignant mesothelial cells in pleural and pericardial fluids. *J. Pathol.*, **160**, 161A.

LOFTUS, B., DERVAN, P., TOBIN, B. & 4 others (1988). AgNOR quantitation and PLAP to identify *in situ* carcinoma of testis. In *Abstracts of XVIII International Congress of I.A.P. and 8th World Congress of Academic and Environmental Pathology*, p.337.

MAURI, F.A., BABARESHI, M., SCAMPIN, S., FERRERO, S. & PERRONE, G. (1990). Nucleolar organiser regions in mucinous tumours of the ovary. *Histopathology*, **16**, 396.

MCNICHOL, A., COLGAN, J., MCMEEKIN, N. & TEASDALE, G.M. (1988). Nucleolar organiser regions in pituitary adenomas. In *Proceedings of the Pathology Society Summer Meeting 1988. J. Pathology*, **155**, 343A.

MIRRE, C. & KNIBIEHLER, B. (1982). A re-evaluation of the relationships between the fibrillar centres and the nucleolus organising regions in reticulated nucleoli: ultrastructural organisation, number and distribution of fibrillar centres in the nucleolus of the mouse Sertoli cell. *J. Cell. Sci.*, **55**, 261.

MORENO, F.J., VILLAMARIN, A., GARCIA-HERDUGO, E. & LOPEZ CAMPOS, J.L. (1989). Silver staining of the nucleolar organizer regions (NORs) in semithin Lowicryl sections. *Stain Technol.*, **63**, 27.

MORGAN, D.W., CROCKER, J., WATTS, A. & SHENOI, P.M. (1988). Salivary gland tumours studied by means of the AgNOR technique. *Histopathology*, **13**, 553.

MURRAY, P.G., BOLDY, D.A., CROCKER, J. & AYRES, J.G. (1989). Sequential demonstration of antigens and AgNORs in frozen and paraffin sections. *J. Pathol.*, **159**, 553.

NAIRN, E.R., CROCKER, J. & MCGOVERN, J. (1988). Limited value of AgNOR enumeration in assessment of thyroid neoplasms. *J. Clin. Pathol.*, **10**, 1136.

NIKICICZ, R.P. & NORBACK, D.H. (1990). Argyrophilic nucleolar organiser region AgNOR staining in normal bone marrow cells. *J. Clin. Pathol.*, **43**, 723.

OCHS, R.L. & BUSCH, H. (1984). Further evidence that phosphoprotein C (110 KD/PI 5.1) is the nucleolar silver staining protein. *Exp. Cell. Res.*, **152**, 260.

OFNER, D., TOTSCH, M., SANDBICHLER, P. & 4 others (1990). Silver staining nucleolar organizer region proteins (AgNORs) as a predictor of prognosis in colonic cancer. *J. Pathol.*, **162**, 45.

OHRI, A.K., HERBERT, A. & ROYLE, G. (1988). Nucleolar organiser regions in breast cancer. *J. Pathol.*, **4**, 347A.

OLERT, J., SAWATZKI, G., KLING, H. & GEBAUER, J. (1979). Cytological and histochemical studies on the mechanism of the selective silver staining of nucleolar organizer regions (NORs). *Histochemistry*, **60**, 91.

PARKER, K.A. & STEITZ, J.A. (1987). Structural analyses of human U3 ribonucleoprotein particle reveal a conserved sequence available for base pairing with pre rRNA. *Mol. Cell. Biol.*, **7** 2899.

PEEBLES, S.A. & MCNICHOL, A.M. (1988). AgNOR counts in rat anterior pituitary corticotrophs following bilateral adrenalectomy. *J. Pathol.*, **155**, 346A.

PLOTON, D., MENAGER, M., JEANNESSON, P., HIMBER, G., PIGEON, F. & ADNET, J.J. (1986). Improvement of staining and in visualisation of the argyophilic proteins of the nucleolar organiser region at the optical level. *Histochem. J.*, **18**, 5.

RAYMOND, W.A. & LEONG, S.-Y.A. (1989). Nucleolar organizer regions relate to growth fractions in human breast carcinoma. *Human Pathol.*, **20**, 1.

REEDER, R.H. (1989). Regulatory elements of the generic ribosomal gene. *Curr. Opinion Cell Biol.*, **1**, 466.

REEVES, B.R., CASEY, G., HONEYCOMBE, J.R. & SMITH, A. (1984). Correlation of differentiation state and silver staining of nucleolar organizers in promyelocytic leukaemia cell line HL-60. *Cancer Genet. Cytogenet.*, **13**, 159.

ROWLANDS, D.C. (1988). Nucleolar organising regions in cervical intraepithelial neoplasia. *J. Clin. Pathol.*, **41**, 1200.

ROWLANDS, D.C., CROCKER, J. & AYRES, J. (1990). An alternative technique for staining nucleolar organizer region associated proteins: use of polyethylene glycol as the protective colloidal developer. *J. Pathol.*, **161**, 349A.

ROSA, J., MEHTA, A. & FILIPE, M.I. (1990). Nucleolar organizer regions in gastric carcinoma and its precursor stages. *Histopathology*, **16**, 265.

ROSA, J., MEHTA, A. & FILIPE, M.I. (1990). Nucleolar organizer regions, proliferative activity and DNA index in gastric carcinoma. *Histopathology*, **16**, 614.

RUSCHOFF, J., BITTINGER, A., NEUMANN, K. & SCHMITZ-MOORMANN, P. (1990). Prognostic significance of nucleolar organizer regions (NORs) in carcinomas of the sigmoid and rectum. *Pathol. Res. Pract.*, **186**, 85.

RUSCHOFF, J. & PLATE, K. (1990). Silver stained structures (AgNORs), their dependence on tissue fixation and abscence of prognostic relevance in rectal adenocarcinoma. *J. Pathol.*, **161**, 89.

RUSCHOFF, J., PLATE, K., BITTINGER, A. & THOMAS, C. (1989). Nucleolar organiser regions (NORs). Basic concepts and practical application in tumour pathology. *Path. Res. Pract.*, **185**, 878.

RUSCHOFF, J., PLATE, K.H., CONTRACTOR, H., KERN, S., ZIMMERMAN, R. & THOMAS, C. (1990). Evaluation of nucleolar organiser regions (NORs) by automatic image analysis: a contribution to standardisation. *J. Pathol.*, **161**, 113.

RUSCHOFF, J., WILLEMARS, S., KLOPPEL, G., ARNOLD, R. & THOMAS, C. (1991). AgNOR and alpha-HCG Bestimmung als Malignantsmarker bei neuroendokvinen Tumoren des Pankreas und Dunndarms. *Verh. Dtsch, Ges. Pathol.*, (in press).

SATOH, K. & BUSCH, H. (1981). Silver staining of phospherine and phosphoturedine in nuclear and other phosphoproteins. *Cell Biol. Int. Rep.*, **5**, 857.

SCHEER, U. & BENEVENTE, R. (1990). Functional and dynamic aspects of the mammalian nucleolus. *BioEssays*, **12**, 14.

SIVRIDIS, E. & SIMS, B. (1990). Nucleolar organiser regions: new prognostic variable in breast carcinomas. *J. Clin. Pathol.*, **43**, 390.

SMITH, R. & CROCKER, J. (1988). Evalulation of nucleolar organiser region-associated proteins in breast malignancy. *Histopathology*, **12**, 113.

SMITH, P., SKILBECK, N., HARRISON, A. & CROCKER, J. (1988). Effect of a series of fixatives on the AgNOR technique. *J. Pathol.*, **155**, 109.

SOOSAY, G., HAPERFIELD, L., PAPADIAKI, L., GRIFFITHS, M. & BOBROW, L. (1989). Evaluation of immunohistochemistry, electron microscopy and silver impregnation of nucleolar organiser regions in the differentiation between malignant mesothelioma, metastatic adenocarcinoma and reactive mesothelial hyperplasia. *J. Pathol.*, **157**, 172A.

SUAREZ, V., NEWMAN, J., HILEY, C., CROCKER, J. & COLLINS, M. (1989). The value of NOR numbers in neoplastic and non neoplastic epithelium of the stomach. *Histopathology*, **14**, 61.

SURESH, U.R., CHAWNER, L., BUCKLEY, H. & FOX, H. (1990). Do AgNOR counts reflect cellular ploidy or cellular proliferation? A study of trophoblastic tissue. *J. Pathol.*, **160**, 213.

TILDSLEY, G., CORBISHLEY, C., SURTEES, P. & RAYTER, Z. (1990). AgNORs, tumour grade, stage and prognosis in colorectal cancer. *Hum. Pathol.*, **160**, 172A.

TRERE, D., PESSION, A. & DERENZINI, M. (1989). The silver stained proteins of interphasic nucleolar organiser regions all parameter of cell duplication rate. *Exp. Cell. Res.*, **184**, 131.

WILLIAMS, M.A., KLEINSCHMIDT, J.A., KROHNE, G. & FRANKE, W.W. (1982). Argyrophilic nuclear and nucleolar proteins of *Xenopus laevis* ooytes identified by gel electrophoresis. *Exp. Cell. Res.*, **137**, 341.

WILLIAMS, R.A., RODE, J., CHARLTON, I.G., JOHNS, L. & MCCARTNEY, A. (1988). DNA content and nucleolar organising regions in ocular melanoma. *J. Pathol.*, **155**, **4**, 342A.

WOOD, A. & EGAN, M. (1989). The silver colloid technique applied to endocervical tissue. *Histopathology*, **15**, 306.

YANG, P., HUANG, G.S. & ZHU, X.S. (1990). Role of nucleolar organizer regions in differentiating malignant from benign tumours of the colon. *J. Clin. Pathol.*, **43**, 235.

YOUNGSHAN, Y. & STANLEY, W.S. (1988). Effect of differentiating agents on nucleolar organiser region activity in human melanoma cells. *Cancer Genet. Cytoget.*, **81**, 253.